# Complete Genome Sequence of a Porcine Epidemic Diarrhea Virus Strain from Vietnam, HUA-14PED96, with a Large Genomic Deletion

**DOI:** 10.1128/genomeA.00002-16

**Published:** 2016-02-18

**Authors:** Se-Eun Choe, Kee-Hwan Park, Seong-In Lim, Van Phan Le, Nguyen Ba Hien, Pham Ngoc Thach, Le Huynh Thanh Phuong, Byung-Hyun An, Song Hee Han, In-Soo Cho, Dong-Jun An

**Affiliations:** a Animal and Plant Quarantine Agency, Anyang, Gyeonggi-do, Republic of Korea; b College of Veterinary Medicine, Vietnam National University of Agriculture (VNUA), Hanoi, Vietnam; c Applied Chemistry and Biological Engineering, Ajou University, Suwon, Republic of Korea

## Abstract

A highly virulent strain of Porcine epidemic diarrhea virus (PEDV) causing severe diarrhea has recently emerged in Vietnam. Genomic sequences from a novel strain, HUA-14PED96, isolated from a Vietnamese piglet with serious diarrhea show relatively high identity with U.S.-like PEDV strains, and have a 72-nt deletion in the open reading frame 1a (ORF1a) gene.

## GENOME ANNOUNCEMENT

Porcine epidemic diarrhea virus (PEDV) belongs to the family *Coronaviridae* and is an enveloped, single stranded RNA virus (1). PEDV was first reported in Belgium and the United Kingdom in 1971 (2), and has since been identified in many European, Asian, and North and South American countries (3). Two genetic types of PEDV are also circulating in the United States; the highly virulent U.S. PEDV strains and the mildly virulent INDEL PEDV strains, the latter of which contain insertions and deletions in the N-terminal region of the spike protein (4, 5). Vietnamese PEDVs detected in 2013 are closely related to Chinese strains (6), and of the two Vietnamese PEDV strains detected in northern and central Vietnam in 2014, one lineage is closely related to Chinese strains and the other to the mildly virulent INDEL PEDV group of strains (7).

In this study, we sequenced the complete genome of a Vietnamese strain of PEDV, HUA-14PED96, and analyzed it to understand the molecular characteristics and diversity of PEDVs in Vietnam.

In October 2014, fecal samples were collected from piglets with watery diarrhea in Hung Yen province, Vietnam. Viral RNA was extracted using the RNeasy minikit (Qiagen, Germany) and cDNA synthesized with the OneStep reverse transcription-PCR kit (Qiagen, Germany). Twenty sets of primers were designed (based on the USA/Iowa/18984/2013 strain) to facilitate amplification of the full-length PEDV genome. PCR products amplified using the primers were cloned into pGEM-T Easy Vector (Promega, USA) and sequenced using an ABI Prism 3730xi DNA sequencer. Multiple PEDV sequences were aligned using the program BIOEDIT 7.053 and Maximum-likelihood estimation, using the general time reversible nucleotide substitution model, was performed using Mega 6.0 software (8).

The length of the HUA-14PED96 genome is 27,966 nucleotides (nt), excluding the 3′ poly (A) tail, and is arranged as follows: A 5′ untranslated region (UTR) comprising 292 nt, open reading frame 1a (ORF1a) and ORF1b encoding a replicase of 20,273 nt, a spike gene comprising 4,161 nt, ORF3 of 675 nt, an envelope gene of 231 nt, a membrane gene of 681 nt, a nucleocapsid gene of 1,326 nt, and a 3′ UTR comprising 334 nt.

The complete genome sequence of HUA-14PED96 shows high nucleotide sequence identity (98.6%) with the highly virulent U.S. PEDV strain (USA/IOWA/18984/2013) and relatively lower identity (97.5%) with a Vietnamese strain (VN/KCHY-310113/2013). Interestingly, sequence analyses of the HUA-14PED96 genome demonstrated a 72-nt deletion in the ORF1a gene, corresponding to a 24 amino acid deletion. Phylogenetic analysis using the nucleotide sequences of the full-length genomes of PEDVs revealed that HUA-14PED96 belonged to the G2 group. The 72-nt deletion could cause a conformational change in the ORF1a protein, resulting in altered antigenicity and immunogenicity of this PEDV variant strain. Further study is required to analyze additional genomic sequences and determine the biological properties of the novel strain, including pathogenicity, tissue tropism, and transmissibility.

### Nucleotide sequence accession number.

The complete genome sequence of PEDV strain HUA-14PED96 has been deposited in GenBank under accession no. KT941120.
